# Don't Look Down: The Limits of Meroscopic Measurement

**DOI:** 10.3389/fresc.2022.873325

**Published:** 2022-03-31

**Authors:** Sue Swenson

**Affiliations:** United States Department of Education, Washington, DC, United States

**Keywords:** outcomes research, human rights, medical model, social model, intersectionality

## Abstract

The essay examines some problems and opportunities of outcomes measures from a philosophical, political, and human rights perspective. Two suggestions for further effort are included: establish a person-centered outcomes research entity to help make outcomes measures more useful in decision-making and use a human rights framework to understand the impact toward which projects and programs aim.

Although he was often mischaracterized as an Aristotelian because he edited the “Introduction to Aristotle” for the University of Chicago Press, Richard McKeon was a strong proponent of pluralism. His commitment was to understand the principles, methods, and interpretations used by any thinker, to understand their work in its own terms, and to escape from the processes of attack and negation that had evolved in academic circles, forcing academics into ever-smaller defensible subject areas. He developed a systemic schema to explain how thinkers might evaluate and appreciate another's project even if it was at odds with their own project. Among the distinctions he made was that between holoscopic first principles, which looked at the part from the perspective of the whole and meroscopic first principles, which looked at the whole from the perspective of the part's simple elements (1). The latter is identified sometimes with Aristotle and the so-called scientific method and involved a separation between the knowers (and their biases) and what is known in a subject matter. I will attempt to identify some of the problems and issues that arise in systems of knowledge about disability when the first principles are universally meroscopic. We need to look up.

Born in medical randomized control trials, expanded in interdisciplinary efforts to prove that social sciences are just as rigorous as medical ones, relied on for decades to help sort proposals for research grants as well as plans and accountability measures for public funding of supports, and required in legislation that authorizes programs, outcome measures are inescapable in modern systems that serve persons with disabilities. They are ripe for rethinking.

Some of the problems of outcomes measures are inherent in the science or the math. Some are moral or ethical. Outcome measures may be the last redoubt of a medical model of disability, rejected by many disability thinkers in favor of the morally acceptable social model. The medical model locates the problems of disability in the person. It is meroscopic. It is supposedly dialectically opposed by the holoscopic social model, which locates the problems of disability in a non-inclusive, non-accommodating society. Even so, most disability services and supports are focused on the individual. This individual focus makes sense if you think of services and supports as something extra to which people are entitled by virtue of their disability. It would be difficult to imagine how the US system of social security disability payments could function without an individual focus. But the focus on the individual can also be a neoliberal fantasy or nightmare of bootstrapping, as if the person alone is responsible, say, for not being able to get or hold a job or for not being “able” to be included and educated in school with everyone else. These are only two examples of activities where the individual clearly must rely on a larger system to become inclusive to achieve goals. But our devotion to individual plans persists. The perseverance of the focus on an individual plan is potentially psychologically destructive to the individual who comes to blame himself or his child for not being able to achieve their goals in the face of systemic denial. That makes it immoral.

Outcome measures are reductive by nature as part of their effort to be “scientific,” reducing the topics of interest in a human life down to measurable facts and indicators, just as a clinical trial of a treatment might measure blood levels of an important marker of progression of disease. But a person is not just an organism, and even medical science is now recognizing that an organism might have fundamental differences from others based on genetic codes. Medical science seems to be recognizing that its abundance of outcome measures does not necessarily help clinicians, patients, caregivers, employers, or others who need to make decisions about treatments. The Patient Centered Outcomes Research Institute (www.pcori.org) was created by Congress in 2010 to address this.

PCORI makes grants of about $2.5 billion per year, and its operating budget is around $105 million with a mission of mediating the utility of NIH research, budgeted at about $52 billion, with around $700 million of that being directed to medical rehabilitation research. The rest of the US federal investment in non-medical rehabilitation, habilitation, special education, and accommodations research is probably <$1 billion dispersed through several departments. A person-centered outcomes research institute in rehabilitation designed to help provide information that supports decision-making of disabled persons and their supporters might be imagined, then, in the $50 million range. Of course, the size of the health care market in the US at about 20% of GDP far outstrips the size of the market for rehabilitation: there is certainly more at stake in healthcare in terms of money.

Because the rehab investigator's points of interest are carefully delineated (meroscopic), rather than taking people as they are in the wild, outcome measures represent a truncated model of responsibility, measuring only the consequences of a program or intervention while ignoring the freedom, knowledge, capabilities, and sense of duty of the participants. Functioning much like genetic codes but with a much more direct and constant impact on the evolution of human society, these could be called the mimetic codes (2). As modern medicine is beginning to understand that outcomes of medical interventions may be mediated by the genome of the person, it may be important for rehabilitation researchers to recognize that compared to a genome, a person's gestalt (or the sum of all of their mimetic evolution) is even more complex and subject to learning and change, and probably more difficult to define.

Outcome measures focus only on the intended results and outcomes of a program without having the capability of identifying or reporting unintended consequences. The unintended consequences of behavior interventions can be severe and lifelong, but outcome measures often frame temporary compliance as success. In fact, the goal of a particular intervention may be defined as compliance with a specific instruction but the lifelong implications of entrained or enforced compliance might well include increased vulnerability, loss of a sense of self, and severely impaired self-efficacy.

Outcome measures of interventions and demonstrations seldom report actual financial results and costs. If a project is designed for a specified cohort but only half of the people identified as the target cohort ultimately participate, then the cost of the intervention is twice what was proposed. Likewise, in-kind contributions and opportunity costs of the intervention are generally not included as inputs or results. If a person spends most of their waking hours in treatment or therapy, what has been lost? Especially for children, the loss of opportunities for free play is not trivial. If an intervention requires a parent or family member to implement the intervention “at home,” the loss to the child may be especially significant. Such a child may cease to see their parent as a comforting and nurturing presence always on their side and instead see them as another therapist with performance demands.

Outcome measures of social interventions focus on the person of interest and seldom consider what happens to others in their circle of support. Did the mother forgo employment to enable her adult son or daughter's independence or employment? Have her human rights been affected? Did a family experience divorce because of the stress of a “therapy” protocol? Is a personal assistant working at minimum wage and without health insurance? Is a caregiver required to ignore OSHA lifting standards at personal risk? Or worse, is the person with disabilities essentially abandoned to an overwhelmed family caregiver who may be depressed or abusive? The “outcomes” of new funding models like self-determination or self-direction require a closer look.

Outcome measures of systems already in place, rather than interventions or research studies, have responsibilities to the public, framed sometimes as diversity or intersectionality. It is possible to argue that in a public system meant to serve “the public,” system outcomes should consist of a sum of outcomes of services and supports provided plus (or minus) the outcomes in the lives of others who received no support or services even though they would be eligible. In other words, an ethical system measures the outcomes for the persons served as well as the persons who should be served. Without this commitment, public services are at risk of overspending on a few people while ignoring many others and counting only the positive outcomes achieved by the few.

Sometimes, too, guardians are praised for the outcomes of their advocacy when they maximize the use of available funds, even when overspending may be negatively impacting the person's ultimate outcomes. This is analogous to a medical patient who does not understand that overtreatment can be as dangerous to their health as under-treatment. For example, if a State requires that all self-directed waiver funds go to direct support wages, a person may be in a position where they are staffed for every waking moment of their day. A man with intellectual disability described this to me as “one person to do the cooking and another to sit on the couch and control the remote.” By the way, this man wanted nothing more than to do his own cooking, as he previously had been employed as a cook. He knew how to make hamburgers the way he liked them, but his funding plan meant he could never have that. He knew what he wanted to watch on TV, but he could not have that either. He enjoyed his solitary time, but that was always denied to him. And meanwhile, while this man is overserved, there are others with the same disabilities whose only supports come from their families as they live their lives on a waiting list. A holoscopic view is needed.

Outcome measures are part of an ongoing effort to construct an evidence base and protocols to guide how a person with a disability might be supported. This is especially obvious in educational environments, where recognized evidence is often limited to published peer-reviewed studies. There are several problems that accrue.

First, demonstrations may be carried out in separate or segregated environments for purposes of keeping “clean” data on individuals (a meroscopic goal). Often, schools attempting to duplicate these outcomes will copy the setting because they are trying to “demonstrate fidelity.” And yet, a segregated environment is a violation of the human rights and the educational rights of the student, which must be prior to any considerations of intervention fidelity. Why should an intervention ever be tested in a segregated environment in the first place?

Second, students who have multiple or complex disabilities—who live their lives three standard deviations from the mean—are not included in statistical studies of interventions. They simply do not fit the definition. Thus, there is no specific evidence for supports that would “work” for them. Unfortunately, the absence of evidence may be interpreted in cash-strapped educational and human services settings as an excuse to do nothing. Worse, artificial intelligence may ignore or misconstrue their existence completely (3). There is often not a recognized floor of standard treatment or standard of care in education or human services as there is in medicine. This should be an ethical requirement and presumption in all systems that serve people with disabilities given that people with the most complex disabilities might appear nowhere in the hierarchy of evidence other than in the foundation of expert opinion and then again in epidemiological (or systems) studies. Without minimum standards of treatment *for all people*, I do not understand how reports of the outcomes of any targeted intervention can be ethical.

Third, educators are often not trained or supported to carry out and report their own outcome measures. If a teacher notices that a child is calm, focused, and happy when included in a small reading group, and tense and lashing out whenever they are taken into the little room for “direct service,” that is an important outcome measure. It should stand up in planning meetings just as well as more incongruent published evidence does. It is important to remember that expert opinion is the foundation of evidence, and teachers and parents are often the only persons who have expertise about a particular child.

Fourth, outcome measures asserting evidence in favor of a particular intervention may be rigged through political processes that organize academic departments or they may slip through the cracks of peer review. No matter how carefully a peer review team is constructed, bad actors can infiltrate and carry their personal animosities or material interests into the review.

A plethora of seemingly positive studies for a single approach may make it seem like this is the only possible intervention. But just because something “works” for a selected sample does not mean it would work for everyone. Likewise, just because one study demonstrates that an approach “does not work” for one or more people does not mean that it cannot work for anyone. Those are both misconstructions of the meaning of statistical studies. And what if a set of studies were all conducted or even funded by persons who engage as professionals in the delivery of that intervention? What if some of them actively agitate to reduce funding to studies of alternative methods, or to deny academic appointments to persons who study alternative methods? This is not, strictly speaking, a problem of outcomes measures alone. It is a problem of research and of universities, and holoscopic. Nevertheless, outcomes can be where bad actors hide.

Fifth, the owners of interventions, whether they can legitimately claim intellectual property or are simply recognized experts in a topic, can use outcome measures in a kind of marketing sleight of hand to convince others that an intervention promises more than it really delivers. Some of these others may be naïve. Some, especially family members, are under enormous pressure or even duress. Some find themselves in a situation where they hope too much or are under pressure to find solutions. These include legislators, administrators of federal, state, and local programs, insurance executives, employers, educators, and families. This marketing is a meta-outcome of outcomes research which provides the tools and rhetoric to people who have their own enrichment as their only goal, or those who believe their own PR. I wish I knew what the answer to this problem could be. As a trained professional marketer, I used to say that you cannot sell a face cream with the same minimal level of evidence that most disability interventions demonstrate. Alas, the US Food and Drug Administration has proven itself quite incapable of regulating even recognized torture perpetrated on people with disabilities in the name of “treatment” with the same brio as it regulates cosmetics.

Outcome measures for human beings are typically not framed in terms of an intervention's likelihood to support or advance the human rights of the person being studied or others who are instrumental. It may be seen as “soft” to do so. Human rights are often reduced to the standards used by an Institutional Review Board where that exists, but these are not likely to be comprehensive enough and they do not touch every situation where outcome measures are used. Indeed, an IRB is more concerned with discovering how the process of a study may itself intrude on human rights, more than it seeks to know whether the ultimate outcome of the study might allow a person to better enjoy or expand their human rights.

A human rights framework is not a requirement of funding designs, but it is easy to see that a human rights framework would provide some of the necessary thinking to compare outcomes across two or more interventions. One intervention may be aimed at creating an outcome of reliable communication support for a person who does not use their voice to speak; simultaneously, another may be designed to help a person practice making sounds that may someday facilitate using their voice to speak. The conflict should be clear: pursuing communication support is a more direct way to achieve self-direction and independence, participation in meaningful education, better health care, the exercise of political, civil, economic, and social rights, and a host of other outcomes that directly impact the human rights of the person.

Perhaps we should make more effort to frame the interventions research we conduct in terms of the human rights the intervention is intended to achieve. Perhaps we can engage in a method of inquiry within an established framework of universal rights consistent with the Universal Declaration of Human Rights (UDHR) and the Convention on the Rights of People with Disabilities (CRPD). Besides being able to explain how the outcomes sought by a research project or program are pertinent to the physical or medical problems faced by the research sample or population, we would be able to discuss not just why we are doing no harm to the persons and their communities but also how these outcomes would help attain or secure their human rights or the human rights of everyone.

None of this is meant to impugn the rigor or intention of social sciences research. People struggle mightily to bring forth new ideas within the strict requirements of government grant applications. It may take more than one effort of inquiry and more than one run at asking why before we arrive at a statement of why a program or intervention supports human rights, or perhaps it will be immediately obvious that there is no connection to human rights at all. If the latter, why should the research or the program be funded with public money?

Sue Swenson and her husband raised three sons, one of whom had multiple and profound disabilities requiring lifelong educational and human services. Sue has served four US presidential administrations in various disability-focused offices, currently on a short-term advisory appointment in special education and rehabilitative services. Sue was educated at the University of Chicago (AB, AM) and holds an MBA from the University of Minnesota. She serves as president of Inclusion International and treasurer of the International Disability Alliance.

The author wrote this commentary outside the scope of their employment with the U.S. Department of Education. The contents of this publication do not necessarily reflect the views or policies of the U.S. Department of Education nor does mention of trade names, commercial products, or organizations imply endorsement of same by the U.S. Government.

## Data Availability Statement

The original contributions presented in the study are included in the article/supplementary material, further inquiries can be directed to the corresponding author.

## Author Contributions

The author confirms being the sole contributor of this work and has approved it for publication.

## Author Disclaimer

The author wrote this commentary outside the scope of their employment with the U.S. Department of Education. The contents of this publication do not necessarily reflect the views or policies of the U.S. Department of Education.

## Conflict of Interest

The author declares that the research was conducted in the absence of any commercial or financial relationships that could be construed as a potential conflict of interest.

## Publisher's Note

All claims expressed in this article are solely those of the authors and do not necessarily represent those of their affiliated organizations, or those of the publisher, the editors and the reviewers. Any product that may be evaluated in this article, or claim that may be made by its manufacturer, is not guaranteed or endorsed by the publisher.
